# Motivation Through Treatment: How Lecanemab Initiation Facilitated Alcohol Cessation and Cognitive Stability in a Patient With Early Alzheimer’s Disease

**DOI:** 10.7759/cureus.90907

**Published:** 2025-08-24

**Authors:** Kentaro Uchida, Jumpei Maruta, Hideo Kurozumi, Koki Inoue

**Affiliations:** 1 Psychiatry, Osaka City Kosaiin Hospital, Osaka, JPN; 2 Neuropsychiatry, Osaka Metropolitan University Graduate School of Medicine, Osaka, JPN

**Keywords:** early alzheimer’s disease, excessive alcohol consumption, lecanemab, lifestyle modification​, mild cognitive impairment, psychiatric intervention strategies

## Abstract

Excessive alcohol use can complicate the management of individuals in the early stages of cognitive decline, as memory impairment may limit the awareness of harmful habits and reduce motivation for behavioral change. Anti-amyloid therapies such as lecanemab have recently become available for early Alzheimer’s disease (AD), but their use in individuals with a history of heavy alcohol consumption remains uncommon. Furthermore, the combined impact of abstinence and disease-modifying therapy on clinical outcomes is not well understood. We report the case of a woman in her early 80s initially diagnosed with mild cognitive impairment (MCI), who later progressed to mild AD. Despite repeated counseling, she had persistent difficulties reducing alcohol intake. As her cognitive symptoms worsened, psychiatric intervention and inpatient care were needed to achieve alcohol cessation. With structured support and lifestyle changes, including the involvement of her daughter, her daily routine stabilized. Following the initiation of lecanemab therapy, she maintained abstinence, experienced no significant treatment-related adverse effects, and showed no imaging abnormalities on follow-up MRI. Her Mini-Mental State Examination (MMSE) scores improved after the combined lifestyle and pharmacologic interventions. This case suggests that lifestyle stabilization and lecanemab therapy can work synergistically in individuals with early AD and prior excessive alcohol use. The initiation of regular infusions may have provided both therapeutic and motivational support for maintaining abstinence and daily structure.

## Introduction

Lifestyle modifications are essential in the treatment of early Alzheimer’s disease and related cognitive impairment. Risk factors for dementia include physical inactivity, poor diet, smoking, excessive alcohol consumption, and obesity [1,2]. Middle-aged obesity and smoking have been reported to be particularly harmful to cognitive function later in life [2,3]. Patients with mild cognitive impairment (MCI) or dementia may develop anxiety and agitation owing to their awareness of cognitive decline, which can lead to excessive drinking, smoking, and overeating. Psychiatrists play a vital role in changing these lifestyle habits.

Motivation is a key factor in promoting lifestyle modifications [4]; however, memory impairment in MCI or mild dementia may make it difficult to maintain. Unlike patients with moderate or severe Alzheimer’s dementia, individuals with early Alzheimer’s disease (AD) (MCI due to Alzheimer’s disease or mild Alzheimer’s dementia) can still purchase alcohol independently, making interventions for inappropriate alcohol consumption particularly important. These patients may also forget both drinking episodes and motivational discussions that took place with clinicians.

Lecanemab, an anti-amyloid-β antibody, was approved for reimbursement in Japan in December 2023 for the treatment of MCI due to AD and mild early AD [5]. However, unstable lifestyle patterns due to drinking in patients with early AD and excessive alcohol use may hinder adherence to the regular lecanemab infusions required for treatment. Intoxication may also delay the detection of adverse events, such as amyloid-related imaging abnormalities (ARIA), making the initiation of lecanemab treatment more challenging. There are no prior reports on the implementation of lecanemab in patients with early AD and excessive alcohol consumption. Moreover, little is known about how motivational factors and lifestyle stabilization interact with lecanemab therapy to support adherence and outcomes. Additionally, the clinical course of synergistic alcohol cessation and lecanemab therapy remains unclear.

While lecanemab is indicated for patients with MCI due to AD or mild AD who meet specific eligibility criteria, successful initiation and continuation of therapy also depend on non-pharmacological factors. In particular, patients’ expectations, motivation, and stabilization of daily life routines are essential prerequisites for adhering to the regular infusion schedule and for safely managing potential adverse events. This case primarily highlights how such non-pharmacological elements contributed to the successful initiation of lecanemab therapy. Here, we report a case in which psychiatric intervention and lifestyle improvements enabled the initiation and continuation of lecanemab treatment in a patient with early-stage AD and excessive alcohol consumption.

## Case presentation

A woman in her early 80s presented with a history of excessive alcohol consumption, drinking several cans of beer and three glasses of shochu per day (approximately 70 g of alcohol). She had a medical history of hypertension, dyslipidemia, and atrial fibrillation, for which she was taking anticoagulants. She also had lumbar spinal canal stenosis. She was a high school graduate and worked in sales until the age of 65. She had married at 25, divorced at 35, and had one daughter with whom she was living at the time of her initial consultation. She consumed large amounts of alcohol daily and sometimes drank during the day. At approximately 75 years of age, she began exhibiting decreased motivation, stopped performing household chores, and started missing appointments at her orthopedic clinic. In July 2020, the patient was referred to our hospital for an initial psychiatric assessment.

Computed tomography of the head showed bilateral temporal lobe atrophy. Blood tests (Table 1) and cognitive assessments were conducted, and her Mini-Mental State Examination (MMSE) score was 21, leading to a diagnosis of MCI. The patient showed elevated γ-glutamyl transpeptidase (γ-GTP) and triglyceride levels, which was considered to be related to excessive alcohol consumption, and was advised to gradually reduce alcohol intake by incorporating alcohol-free days and refraining from daytime consumption; however, despite ongoing guidance, she struggled to reduce her alcohol consumption, and her cognitive impairment worsened. Consequently, she was hospitalized for alcohol cessation in 2021 before the current evaluation. Her executive dysfunction worsened, prompting a revised diagnosis of mild AD and initiation of donepezil 5 mg daily. With the cooperation of her daughter, she gradually adopted a more structured lifestyle, and although she occasionally relapsed into drinking because of insomnia, habitual heavy drinking ceased.

**Table 1:** Laboratory Test Results at Initial Presentation in July 2020 Values in bold are above normal values.

Laboratory Test	Value	Reference Range
Total Protein (g/dL)	6.1	6.5-8.2
Albumin (g/dL)	3.7	3.8-5.0
Blood Urea Nitrogen (mg/dL)	12.9	8-20
Creatinine (mg/dL)	0.6	0.6-1.1
Blood Glucose (mg/dL)	106	70-109 (fasting)
Total Cholesterol (mg/dL)	143	<200
Triglycerides (mg/dL)	203	<150
Total Bilirubin (mg/dL)	0.66	0.2-1.2
Sodium (mEq/L)	140	135-145
Potassium (mEq/L)	3.9	3.5-5.0
Chloride (mEq/L)	105	98-107
Aspartate Aminotransferase (AST, U/L)	22	13-30
Alanine Aminotransferase (ALT, U/L)	11	7-23
Cholinesterase (U/L)	241	200-450
Gamma-Glutamyl Transferase (γ-GTP, U/L)	203	10-47 (M), 6-30 (F)
Lactate Dehydrogenase (LDH, U/L)	151	120-240
Alkaline Phosphatase (ALP, U/L)	126	100-350
C-Reactive Protein (CRP, mg/dL)	0.04	<0.3
White Blood Cell Count (×10³/μL)	6.7	3.5-9.0
Red Blood Cell Count (×10⁴/μL)	361	380-500 (M), 350–470 (F)
Hemoglobin (g/dL)	12.1	13.5-17.5 (M), 11.5–15.0 (F)
Hematocrit (%)	36.8	40-52 (M), 35–47 (F)
Platelet Count (×10⁴/μL)	33.6	15-35
Folic Acid (ng/mL)	6.7	4.0-20.0
Vitamin B12 (pg/mL)	432	200-900
Vitamin B1 (ng/mL)	29	20-80
Free Thyroxine (Free T4, ng/dL)	1.11	0.8-1.8
Thyroid-Stimulating Hormone (TSH, μIU/mL)	0.631	0.4-4.0

**Figure 1:**
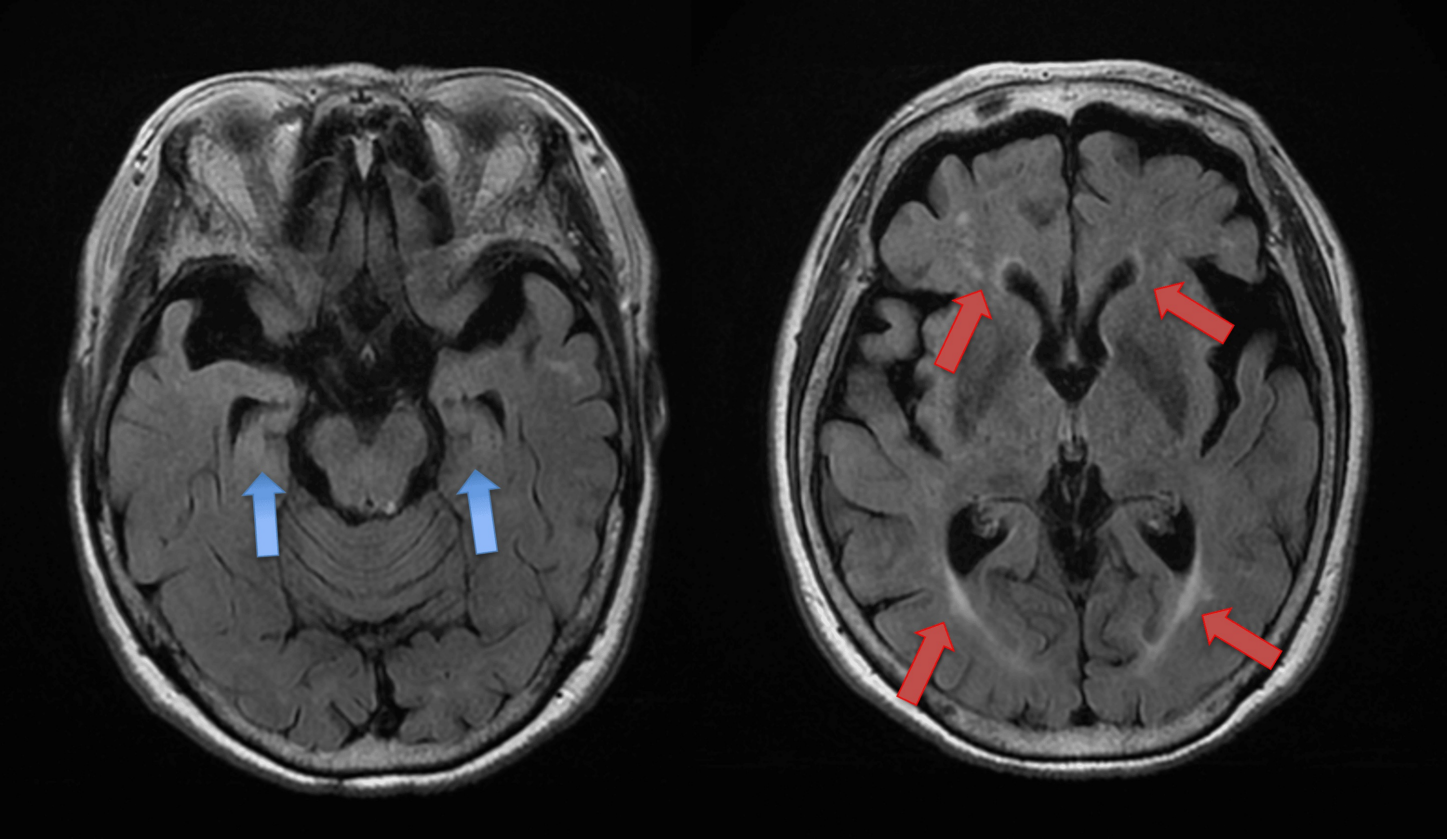
Pre-treatment brain magnetic resonance imaging (MRI) findings Axial T2-weighted fluid-attenuated inversion recovery (T2-FLAIR) magnetic resonance imaging (MRI), obtained prior to initiation of lecanemab treatment, reveals no hemorrhagic lesions or vascular edema, mild atrophy of the medial temporal lobes (blue arrows) and relatively prominent chronic ischemic changes in the periventricular and deep white matter (red arrows).

**Figure 2:**
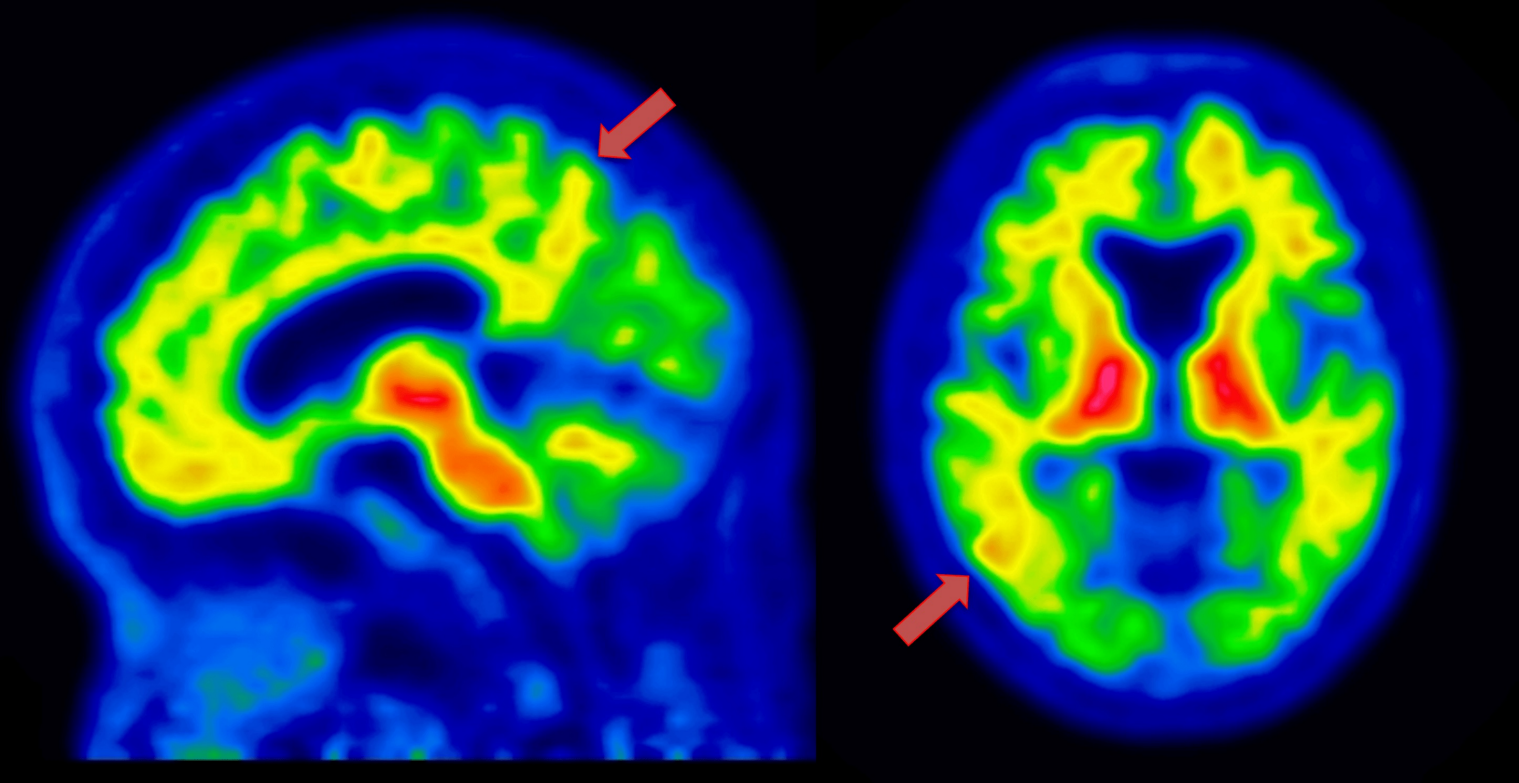
Amyloid positron emission tomography (PET) imaging Amyloid positron emission tomography (PET) shows positive cortical amyloid-β (Aβ) deposition (red arrows), consistent with Alzheimer’s disease pathology.

Her cognitive impairment progressed relatively slowly. In the autumn of 2023, the patient and her daughter expressed a strong desire to receive lecanemab treatment when it became available. By November 2023, her MMSE score was 22, which met the eligibility criteria for lecanemab therapy. Magnetic resonance imaging (MRI) of the head revealed no hemorrhagic lesions or vascular edema that would contraindicate lecanemab use (Figure 1). She began abstaining from alcohol to meet the eligibility criteria and underwent regular infusion. In February 2024, amyloid positron emission tomography, conducted at a collaborating hospital, confirmed amyloid positivity (Figure 2). By March, her MMSE score had improved to 26, and she was deemed eligible for lecanemab treatment. Given that the patient was taking dabigatran, lecanemab was prescribed with caution. After a thorough explanation, the patient and her daughter provided informed consent, and lecanemab treatment commenced in March.

**Figure 3:**
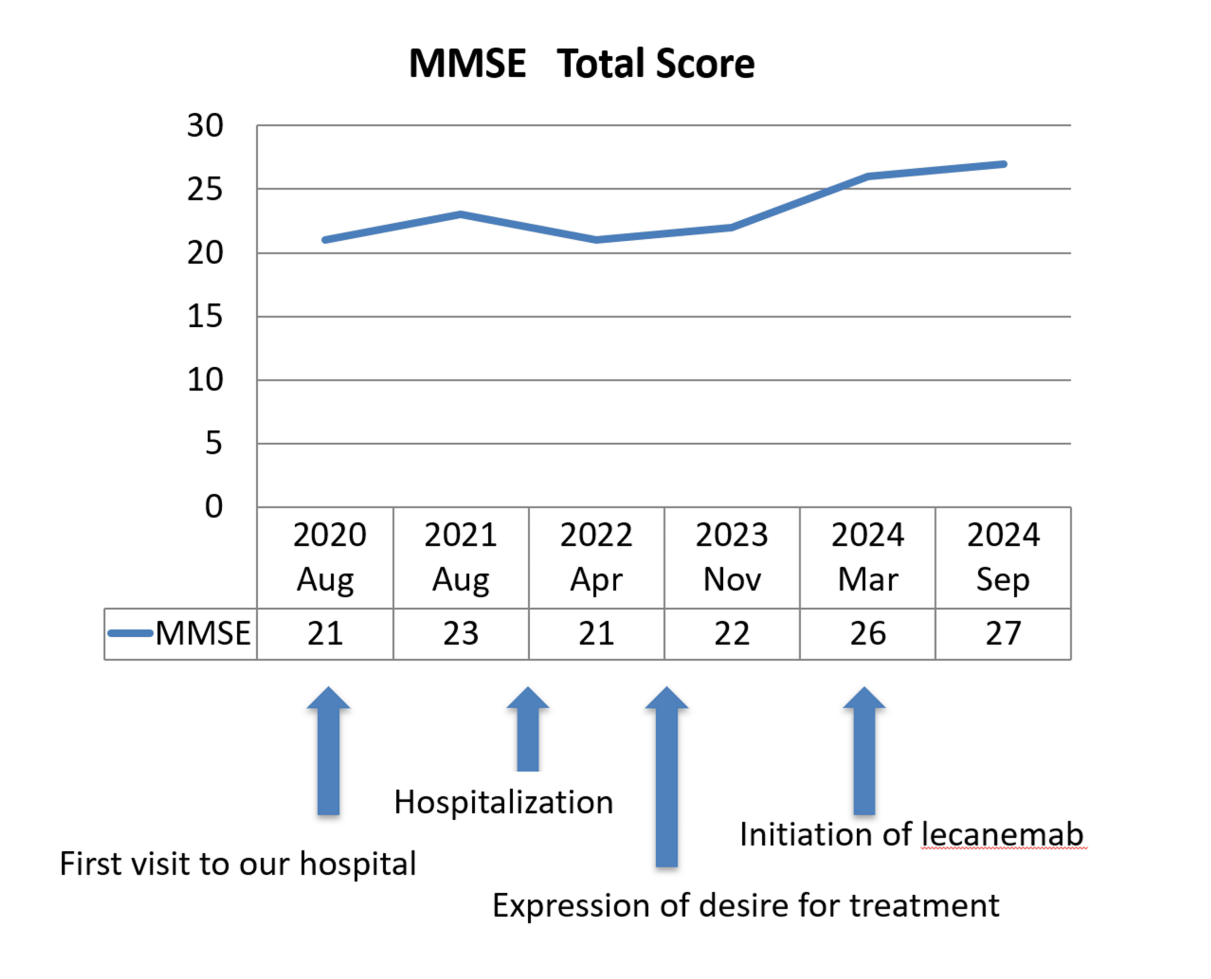
Clinical milestones and Mini-Mental State Examination (MMSE) score progression The Japanese version of the Mini-Mental State Examination (MMSE-J) was used in this study under an official license. The MMSE-J is distributed by Nihon Bunka Kagakusha, and all rights are reserved.

Immediately after the first infusion, the patient experienced chills, which were diagnosed as an infusion reaction. The symptoms gradually subsided with warming using electric blankets and oral antihistamines. Before the second infusion, prophylactic antihistamine premedication was administered to prevent further infusions; subsequent doses did not require antihistamines. Follow-up MRIs performed before the fifth, seventh, and 14th infusions revealed no evidence of ARIA with hemosiderosis/microhemorrhages or edema. Since November 2024, lecanemab treatment has been continued at a follow-up facility, along with regular psychiatric checkups. The clinical milestones, including the hospitalizations, MMSE scores, lecanemab infusions of the patient, are summarized in Figure 3, indicating cognitive stability during lecanemab treatment. Although occasional alcohol consumption associated with insomnia was noted, there was no evidence of excessive drinking, and her daily functioning remained stable.

## Discussion

This case emphasizes the effectiveness of structured lifestyle changes in patients with MCI or AD in improving cognitive function. In this patient, the initiation of lecanemab served as a motivating factor for improving lifestyle habits, including abstaining from alcohol consumption. This improvement subsequently contributed to the stabilization and enhancement of cognitive function. Reports on alcohol-related dementia have shown that abstinence alone can lead not only to stabilization but also to improvement of cognitive function [6]. In the present case, abstinence likely played an important role in maintaining and improving cognition. However, sustained abstinence is notoriously difficult to achieve, with approximately 70% of patients with alcohol use disorder relapsing within one year even when pharmacologic or psychotherapeutic support is provided [7]. In this case, cognitive status was primarily tracked using MMSE scores, while behavioral stabilization was evaluated through clinical observations, abstinence monitoring, and reports from the patient’s daughter. These measures directly reflected the target variables of cognitive stability and motivation for lifestyle change, aligning the observed clinical course with the aims stated in the introduction and the title. Although lecanemab itself does not directly result in immediate cognitive improvement [8], its initiation in this case appeared to motivate abstinence and treatment adherence, which may have synergistically contributed to the observed cognitive benefits.

In Japan, an MMSE score ≥22 is required for lecanemab eligibility. For this patient, the opportunity to receive new anti-amyloid-β therapies such as lecanemab was a powerful motivator for abstinence. Additionally, the biweekly hospital visits required for intravenous infusions helped to structure her routine, reinforcing lifestyle stability. These external frameworks appear to have worked synergistically with psychiatric support to maintain long-term behavioral changes. However, further interventions are required to promote a balanced diet and physical activity, both of which are essential for dementia prevention and cognitive health. Additionally, consideration should be given to securing long-term stability through public long-term care services and social support for such patients to minimize the risk of future decline. This case also illustrates that cognitive stability during lecanemab treatment was not attributable to pharmacotherapy alone but also to the patient’s strong motivation and lifestyle efforts. These factors may be transferable to other patients with early AD who face similar challenges, highlighting the broader clinical applicability of this experience.

## Conclusions

This case highlights how alcohol cessation and lecanemab therapy work synergistically in early AD and MCI. Lecanemab initiation served as a strong motivator for abstinence, which was reinforced by the requirement for regular biweekly infusions; these structured demands supported sustained behavioral changes. Although psychiatric symptoms and lifestyle issues may hinder treatment initiation, timely psychiatric interventions can facilitate therapy and contribute to improved adherence and stability. Since treatment with lecanemab follows a strictly defined protocol for a treatment period of 18 months, it is important to keep patients motivated throughout the treatment period in order to detect side effects and prevent patients from dropping out of treatment or deviating from the protocol; therefore, continuous patient support is required. At our hospital, in addition to the doctor's examination during each biweekly lecanemab administration, skilled nurses with dementia nursing qualifications attempt to determine the patients' living conditions and identify slight changes in their understanding of and motivation for lecanemab treatment from a different perspective from that of the doctor. Treatment with this new drug may reaffirm the importance of traditional post-diagnostic support for dementia.

## References

[1] Agarwal S (2021). Lifestyles and cognition. Integrative Journal of Medical Sciences.

[2] Lee Y, Kim J, Back JH (2009). The influence of multiple lifestyle behaviors on cognitive function in older persons living in the community. Prev Med.

[3] Lo AHY, Pachana NA, Byrne GJ, Sachdev PS (2012). A review of tobacco, alcohol, adiposity, and activity as predictors of cognitive change. Clin Gerontol.

[4] Rubak S, Sandbaek A, Lauritzen T, Christensen B (2005). Motivational interviewing: a systematic review and meta-analysis. Br J Gen Pract.

[5] (2025). “LEQEMBI® Intravenous Infusion” (Lecanemab) approved for the treatment of Alzheimer’s disease in Japan | News Release. https://www.eisai.com/news/2023/news202359.html.

[6] Ridley NJ, Draper B, Withall A (2013). Alcohol-related dementia: an update of the evidence. Alzheimers Res Ther.

[7] Anton RF, O'Malley SS, Ciraulo DA (2006). Combined pharmacotherapies and behavioral interventions for alcohol dependence: the COMBINE study: a randomized controlled trial. JAMA.

[8] van Dyck CH, Swanson CJ, Aisen P (2023). Lecanemab in early Alzheimer's disease. N Engl J Med.

